# Editorial to the Special Issue “Activations of Cadherin Signaling in Cancer”

**DOI:** 10.3390/ijms23137022

**Published:** 2022-06-24

**Authors:** Antonella Tomassetti

**Affiliations:** Unit of Molecular Therapies, Department of Research, Fondazione IRCCS Istituto Nazionale dei Tumori, Via Amadeo 42, 20133 Milan, Italy; antonella.tomassetti@istitutotumori.mi.it

The major object of this Editorial is to briefly put into context the processes, occurring during tumor onset and progression, and the biological mechanisms mediated by cadherins described in the review and research articles included in the Special Issue entitled “Activations of Cadherin Signaling in Cancer”. Indeed, in the last decade or so, some cadherin-associated processes have been investigated more in-depth, allowing the development of some revisited theories to elucidate cancer cell growth and metastasization. In the articles included in this Special Issue several novel and interesting features have emerged that are associated to cadherins expressed in cells from different tumors. 

The cadherin superfamily of cell–cell adhesion molecules comprises 32 members subdivided further into classical type I, type II, 7D and Flamingo cadherins; they are thought to be structural components contributing to the strength and stabilization of the cytoskeleton in cells adherent to each other (reviewed in [1]). In cancer, the expression and function of the most studied cadherins, the E- and N-cadherin, were associated with the transition from a more differentiated phenotype to a more motile, invasive and aggressive phenotype. Since 1991 [2], the E-cadherin has been proposed as a tumor suppressor gene and by in vitro and in vivo models the loss of strong E-cadherin-mediated cell–cell adhesion in cancer cells was proven to be the cause of the acquisition of mesenchymal features, thus leading to metastasis formation. These processes appear very similar to those occurring during tissue development, leading to the epithelial–mesenchymal transition (EMT). EMT is the consequence of a complex transcriptional reprogramming involving epithelial-specific microRNA and mesenchymal-specific transcription factors. As an example shown in this Special Issue, in sporadic intestinal gastric cancer, the CDH1 (the gene encoding for E-cadherin) transcript has been shown to be significantly down-modulated with respect to normal tissue samples [3]. Accordingly, some specific microRNAs, predicted to directly or indirectly regulate CDH1 transcript -miR-101, miR26b and miR-200c-, were also down-regulated in this histotype of gastric cancer. The authors obtained evidence that the transcription factor EZH2 was inversely correlated to CDH1 in the same tumors, arguing for the hypothesis that this transcription factor, regulated by those specific microRNAs, might be responsible for E-cadherin down-modulation in these carcinomas, leading to EMT and progression. 

The switch from E- to N-cadherin has a clinical impact; N-cadherin expression is prognostic in colorectal cancer and is significantly associated with tumor grade, size and nodes, and metastasis stage. Thus, high N-cadherin expression cancer was an independent prognostic factor in both disease-free and overall survival. Looking at the normal epithelium, E-cadherin is differentially expressed by the colonic crypts from the base to the top, where it is necessary for the integrity of the tissue barrier. In colon cancer cells, E-cadherin has been extensively studied with respect to the Wnt/β-catenin pathway activation, which is a driver of tumorigenesis in this epithelium. More recently, E-cadherin stabilized adherens junctions (AJs) resulted to directly interacting with signaling molecules. In this regard, novel molecular mechanisms have been described and unvealnew roles of E-cadherin in the AJs of normal versus cancer cells. Indeed, at the site of the zonula adherens, the apical site of the AJs, the complex of p120-catenin, E-cadherin and PLEKHA7 has been discovered to interact with the microRNA machinery in order to regulate normal epithelial program [4]. In tumor cells, where PLEKHA7 is delocalized or lost, E-cadherin continues to be expressed at the membrane and, while it forms destabilized AJs, acquires the capability of interacting with other signaling molecules, like receptor or non-receptor tyrosine kinases, contributing to pro-proliferative signals. In the transformed context, E-cadherin can also inhibit apoptosis, binding the apoptotic FAS receptor at the site of the AJs, thus interfering with the apoptotic DISC formation [4]. 

In the last decade, EMT has emerged as a reversible process, rather than the final step of cancer progression, in which cadherin-switch as well as cadherin-associated signaling contributes to the biochemical and biological processes caused by cell–cell and cell–environment interactions able to impinge tumor cell phenotype and progression [5]. Furthermore, EMT cannot be always necessary for a tumor to progress, but E-cadherin might acquire pro-metastatic potential through environmental signals by losing its adhesive capabilities but persisting in circulating tumor cells, thus becoming necessary for collective migration and metastatic onset. Indeed, already in 2008, the only presence of the cytoplasmic tail of E-cadherin had been proven to be sufficient for metastatic spreading [6].

Accordingly to the latest observations, the function of membrane proteins, including that of cadherins, can be also regulated by proteases-mediated cleavage. E-cadherin is also shed from the membrane of both normal and transformed cells, creating a soluble 80 KD isoform, which is found in the environment or in the biological fluids. Soluble E-cadherin can activate autocrine/paracrine signals, thus activating or inhibiting signaling pathways. The destabilization of AJs followed by the acquisition of a motile cell phenotype is the most studied mechanism involving soluble E-cadherin in various types of cancer. In breast cancer cells, soluble E-cadherin can mediate the activation of the epidermal growth factor receptor (EGFR) family members, thus up-modulating growth and/or survival pathways [7]. Soluble E-cadherin has also been found to be able to form a complex with the insulin growth factor receptor which, in turn, becomes phosphorylated, leading to the activation of downstream signals. Interestingly, this mechanism also induces the up-modulating expression of specific metalloprotease able to produce increased levels of the soluble E-cadherin, generating an activation loop. So far, the mechanism of cadherin cleavage is known to occur through the action of matrix metalloproteases (MMPs) and disintegrin and metalloproteases (ADAMs). In the present Special Issue, RHBDL2, an intra-membrane serine protease of the Rhomboid family, has been proven to produce the soluble E-cadherin in two in vitro models of prostate and breast carcinoma cells [8]. While this serine protease is also able to cleave the endothelial VE-cadherin, it was not found to be able to produce the soluble N-cadherin, arguing for the hypothesis that the action of the RHBDL2 can trigger other cell- or tissue-specific mechanisms. Indeed, the analyzed prostate cancer cells do not use E-cadherin for cell–cell adhesion but, instead, the production of the soluble 80 KD isoform is positively correlated to the increased migratory capability of these cells. It is of note that in the same prostate cancer cell lines, TNFα increased RHBDL2 expression, E-cadherin shedding and migration. The involvement of the inflammatory cytokine TNFα in the release of E-cadherin has been also observed in human nasal epithelial cells and in chronic rhinosinusitis [9]. These very recent findings might also have some implications in inflammation-associated cancer progression and might suggest the possible exploitation of adjuvant anti-inflammatory therapies to inhibit cadherin release and migration. During this time of a pandemic, it is also worth mentioning that the soluble E-cadherin has been also found to be released upon SARS-CoV-2-infected CaCo-2 intestinal cells [10]. Since high inflammation is induced during SARS-CoV-2 infections, the connection between inflammation and the shedding of E-cadherin by proteases, including RHBDL2, is further reinforced.

Soluble E-cadherin can be also shed from cancer cells in the form of exosome. Indeed, exosomes present in ovarian cancer ascites can utilize the soluble E-cadherin to bind the VE-cadherin of endothelial cells and to promote angiogenesis [11]. Aside from this direct involvement of E-cadherin in signaling through exosome inter-cellular communication, other molecules carried by these particular microvesicles can alter the expression of active proteins present on target cells in order to induce AJ destabilization and cadherin switch occurring during EMT. Wang et al. described in depth the role of exosomes in cancer and their connection with the alteration of cadherin function and expression [12]. 

Among the E-cadherin isoforms and aside from the action of the metalloproteases, it is also worth reporting on a recent 24 KDa E-cadherin soluble variant, produced by the repeated transcription of circular RNA in glioblastoma cells [13]. This newly identified E-cadherin circular RNA has oncogenic properties, since the produced soluble E-cadherin, able to bind EGFR with a unique 14 amino-acid tail, contributes to EGF-independent EGFR/STAT-3 activation and proliferation. These very attractive and novel findings open the way for further experimentation, especially in carcinoma cells, in which cross-talk with EGFR has been already described [14]. 

The emerging role of E-cadherin participating in cancer-associated signaling surely justifies the recent efforts to possibly design a therapeutic approach able to inhibit E-cadherin homophilic interactions. We already reported the characterization of a small peptidomimetic, FR159, identified for its ability to inhibit E-cadherin-mediated cell–cell adhesion [15]. The crystal structure of the E-cadherin–E-cadherin complex with FR159, enabled the assessment of the amino-acids involved in the binding of the peptidomimetics to the E-cadherin X-dimer. This knowledge allowed a virtual screening analysis for the identification of drug-like molecules with increased binding and inhibitory capability of the E-cadherin homodimerization. As reported in the present Special Issue [16], the selected compound, AS11, not only has the highest potency compared to the previously identified FR159 but has been demonstrated here to only interfere with E-cadherin-associated and cancer-related mechanisms, such as the invasion of pancreatic cancer cells grown as spheroids. A particular comment by Delle Vedove et al. merits emphasis: they pointed out that this type of compounds might be especially useful to alter the stability of the E-cadherin-mediated cell–cell adhesion without performing a complete inhibition, likely mimics the earlier steps of the EMT-like process occurring in metastatic tumor cells. On the other hand, the stability of E-cadherin-mediated AJs of circulating tumor cells could be increased by the use of a new anti-E-cadherin monoclonal antibody thus reducing the metastatic potential of cancer cells [17]. Overall, these alternative observations warrant further analysis, possibly in in vitro models, which are more similar to in vivo human tumor cell growth, such as tumor organoids from the different stages of progression or organotypic culture that also allow the preservation of both the tissue architecture and inter-cellular interactions.

Some biological features of E-cadherin can also be found for another type I cadherin, CDH4. Indeed, this cadherin can be co-expressed with E-cadherin in carcinomas, thus contributing to maintaining the AJ structure and stability in transformed cells. On the other hand, CDH4 was found to be expressed in neural tissues as well as in glioma cells, where is necessary for the maintenance of tumorigenic potential by positively contributing to growth and invasion in a murine model. Here, CDH4 was confirmed to be able to also overcome the cell–cell inhibition of proliferation and invasion in in vitro models of human glioma cells, in which the switch from CDH4 to CDH2 occur during progression. In addition, the CDH4 transcript is associated with a worst prognosis [18]. 

Skipping from the type I to the type II classical cadherins, the RGD motif-containing cadherins are now emerging as important contributors in the processes that take place during metastasis formation (for review see [19]). So far, the most investigated type II cadherins in oncology are CDH5 (encoding for the VE-cadherin), CDH6 and CDH17. Although these cadherins have a short cytoplasmic tail, they have recently been shown to bind the β-catenin/p120 catenin/α-catenin complex, thus contributing to the cell cytoskeleton through β-actin, like the type I cadherin. In addition, the presence of the RGD motif allows the binding with integrins and can thus contribute to integrin signaling, responsible for migration and invasion. Casal et al., in their report within this Special Issue, described the interaction of these cadherin with signaling molecules through the RGD motif. In cancer cells, as a result of the binding with other cadherins and the α2β1-collagen IV complex, CDH17 might contribute to both cell–cell and cell–matrix interactions but also to cell proliferation and invasion. CDH17-mediated proliferation can be modulated upon interaction with IkB kinase, thus inducing NFkB activation. In addition, this signaling activation can induce migration and invasion due to the up-modulating expression of the metalloproteases MMP2 and MMP9 [19]. Regarding CDH6, it has been found to be highly expressed in thyroid cancer cells, in which its functionality seems to be related to autophagic signaling, likely involving protein domains different than the RGD domain. Because autophagy in cancer cells can either suppress growth by triggering apoptosis or facilitate tumorigenesis, thus increasing cell growth, the investigation into the role of this latest cadherin in relation to autophagy might open a novel field of investigation [19]. Having these cadherins an RGD motif, still another field of investigation has opened and regards the development of molecules—i.e., peptidomimetics or antibodies—able to inhibit the processes/activations mediated by RGD-containing cadherins to be exploited in the design of novel anti-cancer therapeutic approaches.

One more consideration: an EMT-like process facilitates the early step of metastasis but some epithelial features, including the expression and functionality of E-cadherin, must be maintained to ensure collective migration, cluster proliferation and immune evasion, all processes necessary for metastatic colonization [20]. Furthermore, the above described data reported in this Special Issue also highlight that not only the cadherin-mediated adhesion but cell–cell and cell–matrix communications in general are fundamental mechanisms underlying the neoplastic transformation as well as the processes that occur during the onset of metastases. 
